# Lectuers on Diseases of the Skin

**Published:** 1886-03

**Authors:** Henry Wile

**Affiliations:** Lecturer on Dermatology, Atlanta, Ga.


### LECTURES ON DISEASES OF THE SKIN.

DELIVERED AT THE ATLANTA MEDICAL COLLEGE DURING THE SESSION OF 1885–86. BY HENRY WILE, M. D., LECTURER ON DERMATOLOGY, ATLANTA, GA.

### LECTURE I.

HISTORICAL—GENERAL REMARKS—ANATOMY—EPIDERMIS—CORIUM—SUBCUTANEOUS CONNECTIVE TISSUE—BLOOD VESSELS—LYMPHATICS—NERVES—SWEAT GLANDS.

Stenographically reported for THE ATLANTA MEDICAL AND SURGICAL JOURNAL. By W. K. Tewksbury.

GENTLEMEN—In entering upon the subject of Dermatology, I wish at the outset to impress upon your minds the fact that you are not about to study a class of diseases having an entirely separate and distinct pathology, but that diseases of the skin are subject to the same general pathological laws that may operate in the life-history of any other vital organ of the human economy. Yet the skin, on account of its special anatomy and position, being exposed to external violence, influences of temperature changes, and the presence of parasites is, in addition, subject to certain processes peculiar to itself. Dermatology has made such rapid progress in the last fifty years as to entitle it to recognition as a special branch of medicine. Its development during the last few decades has been remarkable when compared with the condition which characterized its growth during the long stretch of preceding centuries, and a few historical facts gleaned from the annals of medicine may not be out of place at this point.

The oldest record which throws any light upon this subject is found in the Bible. There, in Numbers, Deuteronomy and Leviticus, we find descriptions of leprosy, *zaraath*, which, though brief and obscure, are evidence that this disease was known and recognized in ancient times. The practice of medicine in those days was in the hands of the priests, and in regard to leprosy strict hygienic rules were laid down, and patients were scrupulously isolated to prevent contagion.

Other cutaneous affections are mentioned in the Bible, but from the meagre and unsatisfactory descriptions their identity with modern diseases has not been established. The oldest reliable source from which are derived descriptions of diseases of the skin is in the writings of the Grecian medical observers, notably Hippocrates, who flourished in the year 460 B. C. The father of medicine described a number of diseases under the general name of Exanthemata (Gk. exanthein, to blossom out), including the eruptions of variola, scarlatina, rubeola, etc. He also wrote concerning lepra, pityriasis, psora, herpes, erysipelas, alopecia and other conditions, many of whose names pass current to-day, and may be found in any text-book on dermatology. Hippocrates speculated much concerning the relations between affections of the skin and constitutional disorders or diatheses, attributing the former, for the most part, to changes in the juices of the system; also to influences of climate, diet, etc.

Celsus, fifty years B. C., developed the work of Hippocrates and gave descriptions of additional diseases, such as ulçer, abscess, gangrene, psoriasis, carbuncle, sycosis, clavus, vitiligo and others. The work of Celsus, though describing several new processes, is remarkable for the systematic and complete exposition of the subject as presented in the writings of Hippocrates, and it served as a guide and authority through many subsequent centuries.

Galen, in the second century A. D., commented freely upon the writing of Hippocrates and Celsus, giving details of certain affections called herpes and elephantrasis, also enumerating remedies for erysipelas, lichen, etc.

After Galen nothing of importance was accomplished until the beginning or middle of the eighteenth century. All through the dark ages the above authorities were guides and only occasionally would some new disease be described. The Arabians, Ahron, Rhazes, Avicenna, Avenzoar, Huly Abas, between the sixth and twelfth centuries A. D., made some valuable contributions to the knowledge of dermatology as it then existed, they being the first to describe clearly and definitely variola, rubeola, anthrax, favus, and elephantiasis arabum.

Plenck, of Vienna, published in 1776 a small treaties on cutaneous diseases, in which he classified diseases of the skin according to the nature of the lesion, whether vesicular, papular, tubercular, etc., making fourteen classes. This system was crude and faulty, because there are certain diseases which embrace all of these symptoms, but being new at the time it was adopted by many physicians of prominence.

Willan (1798), an English physician, further perfected the system of Plenck, reducing the number of classes to seven—later to nine.

After Willan several other English, French and German physicians set up classifications from time to time, but most of them fell into disuse when Hebra, in 1845, proposed a classification based upon the pathologico-anatomical characters of the diseases. Of this classification I shall say more subsequently.

To study diseases of the skin properly we must take into consideration the anatomy and physiology of the skin, the general symptomatology, general etiology, diagnosis and treatment.

The skin forms, as you know, a membranous covering of the body, and being extensible and flexible adapts itself to all the movements of the joints. It exhibits in some places a certain roughness, while in other places it is more smooth. We know the skin is more smooth upon the flexor or anterior surfaces of the body than upon the extensor or posterior surfaces. At the different orifices it passes gradually over into the mucous membrane, as at the mouth, the nose, etc., and is completely covered with hair with the exception of the glans penis, eyelids, the palms of the hands and soles of the feet and the extensor surfaces of the third phalanges.

If you notice on the backs of your hands you will see certain markings and lines where the skin is divided into smaller and larger areas. In the palms of the hands and soles of the feet these lines are intensified and form distinct ridges and furrows, and on the flexor surfaces of the terminal phalanges these assume a spiral course. The skin varies in thickness, being thickest in the palms of the hands, soles of the feet and extensor surfaces of the body, and thinnest on the eyelids. Besides forming a covering to the body and protecting the delicate subjacent structures, the skin is the seat of the sense of touch, which is most acute on the ends of the fingers.

We come now to consider the anatomy proper of the skin. It is a complex organ, and is composed of three chief layers—the epidermis, the corium and the subcutaneous connective tissue. Besides these layers there are certain appendages of the skin, such as the sebaceous glands, the sweat glands, the hair and nails, and in addition blood-vessels, lymphatics and nerves. The first or outer layer is the epidermis, also called the cuticle. It is cellular in structure and is divided into two sub-layers: the outer or *stratum corneum* or horny layer ; the inner called *stratum mucosum* or mucous layer—*rete mucosum*, *rete malpighii*. The cells comprising these layers are of an epithelial character.

The outer or horny layer of the epidermis is composed of squamous epithelial cells which are without moisture in the upper strata, dry and closely cemented together, and on section present a fibrous appearance when examined microscopically. The cells composing the upper strata are non-uncleated, flat and thin, while those in the lower strata possess more or less thickness. If a fragment of epidermis is treated with liquor potassæ; the component cells swell and can easily be separated from one another.

The *rete mucosum*, or mucous layer, is composed of cells containing living protoplasm with distinct nuclei. The cells vary in shape; those in the strata next to the corium are cylindrical; those just above are cuboidal and polyhedral, and after this they gradually flatten and assume the characters of the cells composing the horny layer. In the rete there are to be found certain polyhedral cells with serrated edges, known as *prickle cells*. A distinctive test between the cells of the horny layer and those of the mucous layer is the fact that the cells of the former, which are dry and lifeless, with difficulty absorb staining fluids, while the cells of the latter, containing living protoplasm, are deeply stained by these agents.

Besides these two layers of the epidermis there is a layer known as the *stratum lucidum*, which is composed of peculiarly transparent, glistening cells. The transparent condition is supposed to be a state of transformation through which the cells of the mucous layer must pass before becoming corneous. It is regarded by some authorities as a part of the horny layer.

Then, again, in the upper part of the *rete mucosum* there are several rows of granular cells called by some writers *stratum granulosum* or granular layer, because the protoplasm of the cells contain numerous fine granules. This layer is a part of the rete, and is no more entitled to be called a distinct layer than the rows of *prickle cells* to which reference was just made.

The corium is the next layer below the epidermis. If we take a section of the skin and submit it to the action of a solution of liquor potassæ, the epidermis can be lifted from the corium, and in that way a clear view of the form or outline of the upper surface of the latter is obtained, and it presents upward prolongations called the *papillæ of the corium.* The corium is the most important structure of the skin. It is called the *derma*, *cutis vera* or true skin, because all important dermal structures are contained in it. It is composed of two layers, the papillary and reticular layers. The upper or papillary layer contains the finger-like prolongations, or papillæ, and is composed of bundles of connective tissue, entwining so as to form a dense net-work. These papillæ are of two kinds, nerve papillæ and vascular papillæ. The nerve papillæ, which are most numerous on the palms of the hands and tips of the fingers where the sense of touch is most acute, contain each a nerve ending. The vascular papillæ, containing each a loop of blood-vessels, are most numerous over the general surface of the body.

The reticular layer of the corium, also a connective tissue structure, but of much looser texture than the papillary layer, is composed of bundles of connective tissue forming a loose network. The reticular layer passes gradually into the subcutaneous connective tissue layer with no sharp line of demarkation between them. The latter layer is also a connective tissue structure, but the bundles have a much looser arrangement than in the reticular layer, and it contains in addition a deposit of fat which varies in amount according to the habit of the individual. This fat is arranged in larger and smaller lobules, each being composed of a varying number of globules which are nothing more than connective tissue cells that have become inflated with oil. Around each lobule of fat there is a fine plexus of capillaries.

The skin is exceedingly vascular, containing arteries and veins. The cutaneous branches coming up from the subcutaneous structures pass into the skin and the larger arterial trunks are mostly found in the subcutaneous connective tissue where they travel horizontally. From these trunks smaller branches are sent upwards, which subdivide, giving off branches to all the appendages of the skin, and forming the most complicated net-works. The papillary layer is the limit of the cutaneous circulation, as the epidermis is entirely avascular.

The lymphatics of the skin are abundant. Some authors claim that between the connective tissue bundles there are certain lymph spaces, from which the lymphatics take their origin. This, however, is disputed by others. The lymphatics begin in the papillary layer of the corium in fine vessels that empty into larger branches, and these are connected with the still larger branches of the subcutaneous connective tissue which in turn are joined to the lymphatics of the deeper structures.

The skin contains nerves, both medullated and non-medullated. The non-medullated nerves terminate in olive-like bodies found in the subcutaneous connective tissue and known as *pacinian corpuscles*. The medullated nerves, on the other hand, traverse all the layers of the skin, having been traced even between the cells of the epidermis. In the papillary layer they terimnate, in the nerve papillæ, in the form of a coil, known as the *touch corpuscles*, and are most numerous in the ends of the fingers and toes. Besides these nerves we have a certain other system of nerves known as the vaso-motor nerves, which have a connection with the blood-vessels and control their calibre. They are of two kinds—the vaso-constrictors and the vaso-dilators. When a person becomes frightened intensely, the vaso-constrictor nerves are stimulated, producing constriction of the blood-vessels through a contraction of the muscular coats. The blood is driven out from the part thus affected and the individual grows pale. On the other hand, the blush of shame serves as an example of the action of the vaso-dilator nerves. In this case the blood-vessels become dilated and an excess of blood is invited to the part producing undue redness.

Then again we have unstriated muscular tissue in the skin. In the scrotum it forms a distinct muscular layer called the dartos. In connection with the hair there is an important muscular structure called the *arreetor peli* muscle, which, together with the muscular fibres scattered between the bundles of connective tissue of the corium, may by contraction produce that condition of the skin known as "goose flesh." The bundle of muscular fibres is attached to the base of the hair follicle, and passing obliquely towards the surface is lost in the upper layers of the corium. By contraction it causes the hair to assume an erect position.

In the cells composing the lowest layers of the *rete mucosum* there may be found a varying amount of pigment which determines the complexion of the individual. If the pigment is very extensive you see a brunette type ; if scanty, a blonde type. In the negro the cells contain a large amount of pigment—in fact, the whole *rete mucosum* is more or less stained with pigment granules.

We come now to speak of the glandular apparatus of the skin. There are two kinds of glands, the sweat glands and the sebaceous glands. The sweat glands are situated in the subcutaneous connective tissue. They are tubular glands, and consist of a coil of tubing having a duct or outlet, which passes through the different layers of the skin in a wavy manner, and, when reaching the horny layer of the epidermis, assumes a corkscrew-like course. The sweat glands are surrounded by a dense plexus of blood-vessels from which the cells of the sweat glands secrete the sweat. These glands, as I said, are tubular, and a very simple structure, being composed of a basement membrane of connective tissue upon which is a single layer of columnar or cuboidal epithelial cells, and around the coil and the duct is a small amount of unstriated muscular fibre. The sweat glands differ as to number in different parts of the body. They are most numerous on the palms of the hands and soles of the feet, and it is estimated that on a square inch in the palm of the hand there are twenty-seven hundred and thirty-six. It is furthermore estimated by some authorities that if the sweat glands of a single individual were all drawn out and connected together so as to form one tube, it would be eight miles long. So you can imagine what an enormous secreting surface these glands have.

